# The Emotional Modulation of Facial Mimicry: A Kinematic Study

**DOI:** 10.3389/fpsyg.2017.02339

**Published:** 2018-01-18

**Authors:** Antonella Tramacere, Pier F. Ferrari, Maurizio Gentilucci, Valeria Giuffrida, Doriana De Marco

**Affiliations:** ^1^Lichtenberg-Kolleg - The Göttingen Institute for Advanced Study, The German Primate Center Cognitive Ethology Lab, Leibniz Institute for Primate Research, Georg-August-Universität Göttingen, Göttingen, Germany; ^2^Unità di Neuroscienze, Dipartimento di Medicina e Chirurgia, Università degli Studi di Parma, Parma, Italy; ^3^Istituto di Neuroscienze–Consiglio Nazionale delle Ricerche (Sede di Parma), Rome, Italy

**Keywords:** basic emotion theory, embodiment, facial mimicry, lip kinematics, mouth gesture, emotional valence, facial expression

## Abstract

It is well-established that the observation of emotional facial expression induces facial mimicry responses in the observers. However, how the interaction between emotional and motor components of facial expressions can modulate the motor behavior of the perceiver is still unknown. We have developed a kinematic experiment to evaluate the effect of different oro-facial expressions on perceiver's face movements. Participants were asked to perform two movements, i.e., lip stretching and lip protrusion, in response to the observation of four meaningful (i.e., smile, angry-mouth, kiss, and spit) and two meaningless mouth gestures. All the stimuli were characterized by different motor patterns (mouth aperture or mouth closure). Response Times and kinematics parameters of the movements (amplitude, duration, and mean velocity) were recorded and analyzed. Results evidenced a dissociated effect on reaction times and movement kinematics. We found shorter reaction time when a mouth movement was preceded by the observation of a meaningful and motorically congruent oro-facial gesture, in line with *facial mimicry* effect. On the contrary, during execution, the perception of smile was associated with the facilitation, in terms of shorter duration and higher velocity of the incongruent movement, i.e., lip protrusion. The same effect resulted in response to kiss and spit that significantly facilitated the execution of lip stretching. We called this phenomenon *facial mimicry reversal effect*, intended as the overturning of the effect normally observed during facial mimicry. In general, the findings show that both motor features and types of emotional oro-facial gestures (conveying positive or negative valence) affect the kinematics of subsequent mouth movements at different levels: while congruent motor features facilitate a general motor response, motor execution could be speeded by gestures that are motorically incongruent with the observed one. Moreover, valence effect depends on the specific movement required. Results are discussed in relation to the Basic Emotion Theory and embodied cognition framework.

## Introduction

In human and non-human primates, gestures are important channels of communication and, within a social context, they are produced either *to initiate* or *to respond* to a social exchange.

The production of one specific gesture depends on the evaluation by the subjects of the on-going social interactions, which in turn rely on the integration between specific sensorial and motor processes. An increasing body of evidence has shown that the perception of various types of manual and facial gestures has a significant influence on the ability to perform correlated actions, and on the parameters associated to their execution (for an extensive discussion of this topic see Engel et al., 2016). As for example, perceiving a manual gesture or a word affected the simultaneous (Bernardis and Gentilucci, 2006; Gentilucci et al., 2006) or subsequent (De Marco et al., 2015) pronunciation of a meaningful word or the execution of a hand movement. Specifically, vocal and kinematic parameters are modulated by the semantic congruence or incongruence between the gestures and words meaning (De Marco et al., 2015).

As for manual gestures, individuals often exhibit (overt or covert) changes in their own facial movements in response to the perception of others' facial expressions. In particular, if such motor responses are similar to those observed they reflect the phenomenon named facial mimicry (Dimberg et al., 2000; Stel and Vonk, 2010; Tramacere and Ferrari, 2016). More specifically, through electromyographic studies (EMG), the activity of facial muscles has been recorded during passive observation of emotional facial expressions (Dimberg, 1990; Dimberg et al., 2000; Sato and Yoshikawa, 2007). It has been found that during the observation of happy face, the muscle involved in the lifting of the lip corners (i.e., *zigomatycus majors*) is activated, while the *corrugator supercilii*, involved in the corrugation of the eyebrows and the formation of wrinkles in the middle of the forehead area, is activated during the observation of a sad face. Interestingly, some EMG studies suggest that the perception of happy face (i.e., smile) produces a stronger effect on the excitation of the facial muscles of the observer, while an angry face produces a more subtle response (Rymarczyk et al., 2016). Further, individuals seem to mimic congruent emotional facial expressions more easily than incongruent ones. Specifically, participants mimic smiling expressions more efficiently and for a longer time in response to a happy facial expression compared to a sad one, and the same is true in the opposite condition (Niedenthal et al., 2001).

Although these studies have investigated the activation of facial muscles during observation of emotional facial stimuli (Korb et al., 2016; Rymarczyk et al., 2016), the variations of kinematics parameters associated with the observations of different types of emotional expressions has not yet been satisfactorily inquired. In particular, the effect of the interactions between motor features and emotional valence on the facial execution of the perceiver is still unknown, making unclear how the observation of various oro-facial gestures communicating different emotional characters would affect the kinematic response of the perceiver.

Specifically, our study aims at determining how the perception of positive or negative oro-facial gesture (i.e., smile, anger-mouth, kiss, or spit) would affect the kinematics parameters of a congruent or incongruent subsequent movement, such as stretching and protruding lips. To this purpose, six dynamic stimuli, showing different positive, negative (meaningful), and neutral (meaningless) oro-facial gestures were presented to participants, who were required to perform two target movements in response to the observed stimuli (i.e., lip protrusion and lip stretching) characterized by having compatible or incompatible motor features with the observed mouth expression.

Considering that anatomically distinct motor areas control the muscles of the upper and lower parts of the face (Asthana and Mandal, 1997), facial expressions engage to a different extent eye-nose and mouth regions, requiring distinct patterns of activity distributed across multiple face motor areas. In order to disambiguate the modular effects of different kinds of mouth expressions, in this study we decided to focus only on the perception and execution of movements performed with the mouth, and on specific positive and negative emotions associated to its configuration. We hypothesized that both motor features (mouth aperture vs. mouth closure) and valence (neutral, positive, or negative) of the facial gestures could influence motor planning and execution of the movements performed by the participants; therefore we measured both Response Times (RTs) and kinematic parameters [Movement amplitude (MA), Movement duration (MD), Mean lip velocity (MV) of the lip movements]. These parameters represent landmarks which characterize the time course of the movement: by measuring them, we determined spatial and temporal variation of the lip movements in relation to the experimental stimuli.

**Figure 1:**
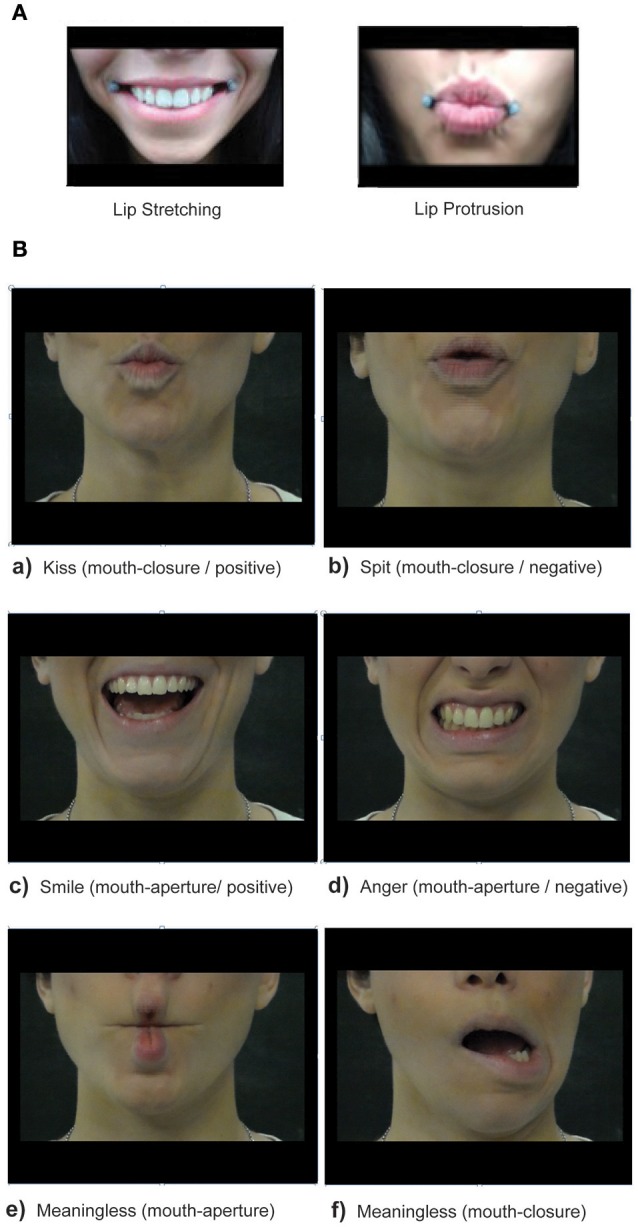
**(A)** Movement tasks. The figure depicted an example of the execution of the Lip Stretching (at the left) and Lip Protrusion (at the right) movements that participants had to perform in response to experimental stimuli. **(B)** Experimental Stimuli. In the upper part are presented mouth-closing orofacial gestures with positive **(a)** and negative **(b)** value. At the center, positive **(c)** and negative **(d)** mouth-opening orofacial gestures are presented. Meaningless closing-mouth **(e)** and opening-mouth **(f)** gestures are depicted at the bottom. The same stimuli executed by a male actor were presented during the experiment.

Previous studies (Hess and Blairy, 2001; Sonnby-Borgström, 2002; Sato et al., 2008) have limited their investigations to the understanding of congruence and consistency between covert facial muscles activation and emotions perception in typical adults, together with an analysis on the capacity to recognize emotions. Although these studies have not disambiguated between the motor patterns of overt facial movements (e.g., mouth-opening or mouth-closing) in response to different types of facial expressions (e.g., positive and negative emotional stimuli), they all converged on the interpretation that the perception of emotional expressions enhances the activation of muscles involved in the same motor program. As consequence, we predicted that the kinematics parameters associated to lip stretching, which resembles a motor pattern of aperture (see Figure 1A) would have been magnified by the observation of mouth-aperture (i.e., smile and anger-mouth), and interfered by mouth-closure gestures (i.e., kiss and split). We also predicted that the opposite was valid for lip protrusion, which resembles a pattern of closing (Figure 1A). Specifically, we expected that, if participants executed a movement that was similar to the observed gesture (e.g., executing a lip protrusion movement after observing mouth closing), the execution of the action would be facilitated, resulting in faster reaction times (RTs), and/or shorter duration of the movement (MD) and/or higher velocity (MLV) and/or longer trajectory (MA); on the contrary, a movement interference would be evidenced by longer reaction times, and/or longer duration, and/or lower velocity, and/or shorter trajectory.

We further hypothesized that even meaningfulness could influence motor response: in fact, similarly to manual gestures (see De Stefani et al., 2013), some oro-facial gestures convey a positive or negative valence. In line with the previous literature (Bourgeois and Hess, 2008; Rymarczyk et al., 2016) we predicted that positive valence (e.g., smile and kiss) facilitates conspecifics interaction, speeding the planning and the execution of the participants' movement, while a negative valence (e.g., angry-mouth and spit) interferes with the response. Furthermore, in order to investigate a possible interaction between motor features and emotional valence on motor response, gestures associated with different emotions were presented either in a congruent (where the motor pattern of the gesture was compatible with that of the response) or in an incongruent condition (opposite motor patterns).

We finally verified whether facial gestures associated with basic emotion expressions such as happiness or anger (Ekman, 1992) produce different effects on the observer compared to gestures which communicate an emotional state by mean of an instrumental action, i.e., kiss and spit. We discussed our results in the framework of Basic Emotion Theory (BET), trying to disambiguate the effect of basic (i.e., smile and angry-mouth) and non-basic (i.e., spit and kiss) emotional expression on different opening and closing lip movements.

## Materials and methods

### Participants

One sample of 14 participants (7 females and 7 males, mean age of 22 ± 2.3 years) took part in the experiment. The participants were right-handed (according to Edinburgh Handedness Inventory, Oldfield, 1971) and Italian native speakers. They had normal or corrected-to-normal vision and no history of neurological or psychiatric disorder; The Ethics Committee of the Medical Faculty at the University of Parma approved the study. The experiment was conducted according to the principles expressed by the Declaration of Helsinki. All the participants in the present study provided written informed consent.

### Apparatus, stimuli, and procedure

The participants sat comfortably in front of a table, maintaining their lips closed. The monitor of a PC (19-inch LCD) was placed on the table plane, 70 cm distant from the participant's forehead. It was set at a spatial resolution of 1,024 × 768 pixels and at a temporal resolution of 60 Hz. The dynamic stimuli presented consisted of video clips during which a male or female actor executed an oro-facial gesture. The mouth movements could be meaningful, expressing an universally recognizable emotional message, or meaningless. The emotional valence conveyed by meaningful stimuli could be positive (kiss or smile) or negative (spit or anger, see Figure 1B). Furthermore, they could be characterized by a mouth-aperture movement (i.e., smile and anger) or a mouth-closure movement (i.e., kiss and spit). Meaningless gestures depicting mouth aperture or closure movement without specific valence were included to match with meaningful stimuli as control. All the videoclips showed only the bottom part of actors face, in order to exclude any effect related to the gaze or upper face.

**Figure 2:**
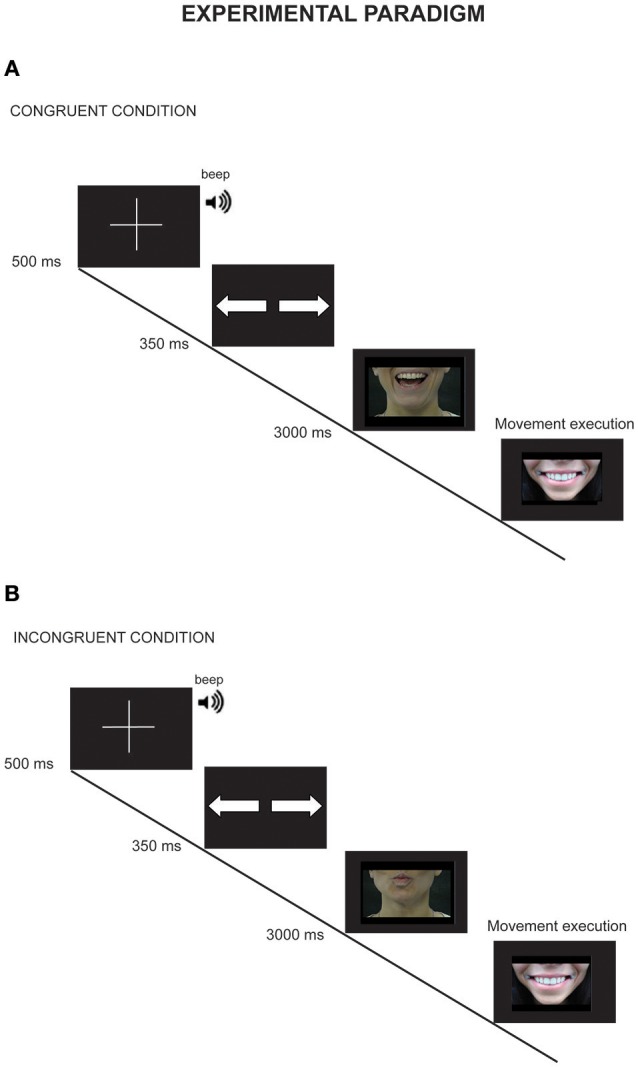
Experimental paradigm. Panel **(A,B)** reported an example of congruent **(A)** and Incongruent **(B)** trials **(A)**. The trial started with a fixation cross (500 ms duration), followed by a cue (diverging arrows, 350 ms duration) that instructed the subject about the movement to perform. After the cue, the video stimulus was presented and the subject started the movement after the end of the video. In this case a mouth-opening gesture was followed by a lip-stretching movement. **(B)** The sequence is the same of that reported in **(A)**. However, in this case, the requested movement was incongruent respect to the visual stimuli.

Stimuli were validated on the basis of the results of a task carried out on a separate sample of 30 volunteers. The task was to judge the meaning and valence of the oro-facial gestures presented by videos, assigning a score from 1 to 6 according to the emotional valence (score 1–2 negative, 3–4 neutral, 5–6 positive). The mean scores of negative and positive (meaningful) and neutral (meaningless) gestures were 1.8, 4.5, and 3.0. ANOVA results showed that they significantly differed from each other [*F*_(2, 18)_ = 14.79, *p* < 0.00001, *post-hoc p* < 0.001]. All the meaningless stimuli were correctly identified as postures without a well-known meaning and no emotional significance (Mean percentages of correct hits: Spit gesture = 93% Kiss gesture = 93% Smile gesture = 100% Anger gesture = 93% Meaningless Aperture gesture = 86% Meaningless Closure gesture = 80%). In total, four video of negative gestures, four videos of positive gestures, and four videos of meaningless gestures were presented in the experimental task, equally represented by male and female actors. Participants' task was to perform a lip movement after the observation of the stimuli. Each trial started with a picture of a fixation cross (500 ms duration) followed by a visual cue where a picture of two arrows instructed the participant about the movement he/she had to perform in the subsequent task (preparation-phase, see Figure 2). The arrows informed the participants on which of the two possible movements they had to execute after the presentation of the oro-facial gesture: lip stretching, indicated by divergent arrows, or lip protrusion, indicated by convergent arrows. A correct lip stretching movement required extending the lips and contemporarily opening the mouth as extensive as possible (Figure 1A); lip protrusion required to close the lips and protrude them forward at the maximum distance (Figure 1A). After the cue presentation, one of video stimuli was presented; the video lasted 3,000 ms, and at the end a still frame showing the actor with a neutral face with closed mouth was shown. Participants were instructed to maintain their lips closed and in a neutral position until to the still face appeared; then, they had to execute the movement previously planned as accurate and fast as possible (execution phase, see Figure 2), and then returned to the starting position. Each stimulus was coupled with a congruent or incongruent movement cue: in the case of congruency, the motor pattern of the observed movement corresponded to that of the executed movement (e.g. lip stretching movement that followed the observation of a mouth-aperture gesture). A total of 120 trials were run (10 repetition for each couple oro-facial gesture—lip movement randomly presented) divided in two blocks. In addition, 20 baseline trials (10 at the beginning of the first block and 10 at the end of the second block) were administered in order to exclude any kinematic effect not related to the experimental variables. During baseline trials participants were instructed to simply perform lip stretching or lip protrusion movement (10 trials for each movement) in response to the cue presented before the still picture of the neutral face of the actor.

### Lip movement recording and data analysis

The lip kinematics during lip movement execution was recorded using the 3D-optoelectronic SMART system (BTS Bioengineering, Milano, Italy). This system consists of six video cameras detecting infrared reflecting markers (spheres of 5-mm diameter) at a sampling rate of 120 Hz. The cameras were positioned around the recording space so that each marker could be detectable from different space angles. The recorded images are integrated each other and visualized on SMART Tracker software (BTS Bioengineering, Milano, Italy) for inspection. Then, data were exported in an output file which reported the three-dimensional (x, y, z) spatial coordinates of each marker at each time frame. Spatial resolution of the system is 0.3 mm; this value was used as criterion also for smoothed data (see beyond). The infrared reflective markers were attached to the right and left side of participant's lip. The data of the recorded movements were analyzed using homemade scripts developed using MATLAB version 7.7 (R2008b). Recorded data were filtered using a Gaussian low pass smoothing filter (sigma value, 0.93). The time course of lip opening and closing was automatically calculated by MATLAB programming the script using the following criteria: the beginning of the movement was considered to be the first frame in which the distance between the two markers placed on the lips increased and decreased more than 0.3 mm (spatial resolution of the recording system, i.e., minimal displacement greater than noise that the system is able to record) with respect to the previous frame. The end of the movement was the first frame in which the distance between the two lips increased/decreased <0.3 mm with respect to the previous frame. In addition, each trial was then visually inspected to check for corrected start-end frame identification, movement artifacts, or errors. Trials with artifacts or errors were discarded for subsequent analysis.

Considering that the experimental task required moving the lips along the horizontal plane, only horizontal lip movements were analyzed. We measured for statistical analysis the following parameters: RTs (Response times), MA (movement amplitude), MD (Movement Duration), and MLV (Mean Lip Velocity). MA for lip stretching was calculated as a vector that represents the difference between the minimal and the maximal distance values along the three axes between the markers in the movement time-window; MLV was calculated as the ratio between maximal lip aperture and time to maximal lip aperture.

The mean values of all the parameters were submitted to a series of a 2 × 2 × 3 repeated-measure ANOVAs with TASK (lip protrusion/lip stretching), GESTURE MOVEMENT (mouth aperture/mouth closure), and VALENCE (neutral/negative/positive) as within subject factors. Concerning kinematic variables, to further interpret the results and discern the gesture effect form task effect, we administered additional *t*-tests in order to compare the mean values of the kinematic parameters measured in response to each experimental stimuli with those of the corresponding baseline trials, where no gesture was observed.

All *post-hoc* comparisons were carried out using Duncan test. Significance was established in all analyses at *p* = 0.05. Sphericity of the data was verified prior to performing statistical analysis (Mauchly's test, *p* > 0.05). All variables were normally distributed as verified performing Kolmogorov–Smirnov Test (*p* > 0.05). Two participants were excluded from the analysis because their mean values of Movement Duration and Mean Lip Velocity resulted as big outliers compared to the sample mean (<3 *SD* compared to sample mean).$\eta_{\text{partial}}^{2}$ was calculated as a measure of effect size.

Mean values and Standard errors of RTs and kinematic parameters for each experimental condition are reported in Table 1. A summary description of all the major significant comparisons and effects is presented in Table 2.

**Table 1:** Mean values (M) and standard errors (SE) of response times and kinematic parameters recorded during the execution of lip protrusion and lip stretching movements for all the experimental conditions.

	**Aperture gesture**						**Closure gesture**					
	**Meaningless**		**Negative**		**Positive**		**Meaningless**		**Negative**		**Positive**	
	**M**	**Es**	**M**	**Es**	**M**	**Es**	**M**	**Es**	**M**	**Es**	**M**	**Es**
**LIP PROTRUSION**												
Response Times—RTs (ms)	449.36	32.60	414.63	18.61	421.47	29.50	448.88	29.76	353.45	19.83	370.26	34.38
Movement Duration—MD(ms)	320.74	26.65	301.78	18.82	276.09	14.95	293.96	13.68	316.38	18.78	321.80	20.40
Mean Lip Velocity—MLV(mm/s)	40.72	3.67	41.47	3.21	44.13	3.22	41.81	2.49	38.68	2.67	39.75	3.16
Movement Amplitude—MA(mm)	12.20	0.67	12.00	0.53	11.80	0.60	12.03	0.55	11.88	0.61	12.20	0.57
**LIP STRETCHING**												
Response Times—RTs (ms)	472.70	26.22	446.60	27.72	402.22	22.10	515.44	33.03	425.75	25.79	430.35	35.88
Movement Duration—MD (ms)	330.92	24.87	360.07	27.91	335.42	26.57	350.42	29.32	327.03	25.15	335.08	24.51
Mean Lip Velocity—MLV (mm/s)	32.38	5.42	29.49	4.69	32.33	5.08	30.82	4.81	31.38	5.23	31.63	5.32
Movement Amplitude—MA (mm)	10.47	1.45	10.42	1.42	10.54	1.45	10.58	1.47	9.97	1.49	10.13	1.49

**Table 2:** Results summary.

	**Lip Protrusion Task**	
	**Significant comparisons**	**Gesture movement and valence effects**
Response Times – RTs	Closure gestures (positive and negative) < aperture gestures (positive and negative) Meaningful gestures (positive and negative) < meaningless gestures	Main effect of gesture movement Main effect of valence (both positive and negative)
Movement Duration—MD	Smile gesture < kiss gesture. Spit gesture and meaningless aperture Smile gesture < baseline Meaningless closure < baseline	Interaction effect of valence and gesture movement Facilitation effect of positive valence in case of incongruent gesture movement Facilitation effect of gesture movement
Mean Lip Velocity – MLV	Smile gesture > kiss gesture and spit gesture Smile gesture > baseline	Interaction effect of valence and gesture movement Facilitation effect of valence (positive) in case of incongruent gesture movement
Movement Amplitude – MA	No significant comparisons	No significant effects
	**Lip Stretching Task**	
	**Significant comparisons**	**Effects**
Response Times—RTs	Meaningful gestures (positive and negative) < meaningless gestures Meaningless closure > baseline	Main effect of valence (both positive and negative) Interference effect of gesture movement
Movement Duration – MD	Kiss gesture < baseline Spit gesture < baseline	Facilitation effect of valence (either positive or negative) in case of incongruent gesture movement
Mean Lip Velocity—MLV	No significant comparisons	No significant effects
Movement Amplitude—MA	No significant comparisons	No significant effects

*Significant results of the ANOVA are described for each dependent variable (Response Times and Kinematic Parameters). Significant main or interaction effects are reported. We have referred to facilitation or interference effects only in case of significant comparisons with respective movement baseline*.

## Results

### RTs

**Figure 3:**
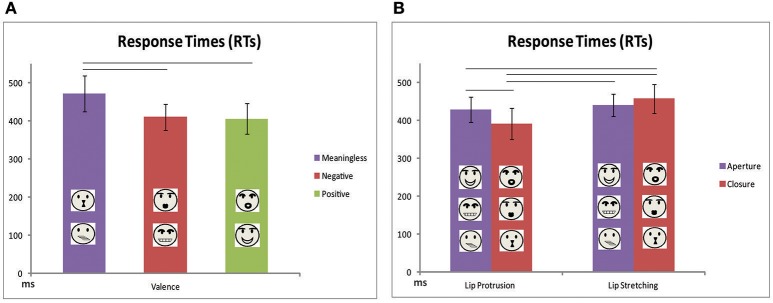
Mean values of Response Times for both lip protrusion and lip stretching movements for the stimuli categorized as meaningless, negative and positive **(A)** and as aperture and closure **(B)**. Other conventions as in Figure 5.

The results of ANOVA showed a significant main effect of factors TASK [*F*_(1, 11)_ = 5.6, p = 0.037, $\eta_{\text{partial}}^{2}$ = 0.34] and VALENCE [*F*_(1, 11)_ = 8.6, *p* = 0.002, $\eta_{\text{partial}}^{2}$ = 0.44, Figure 3A]. Participants started to move earlier when they have to perform a lip protrusion instead of a lip stretching movement. Concerning the VALENCE effect, *post-hoc* analysis revealed that for both tasks participants moved faster after the observation of a meaningful gesture (either a mouth-aperture or mouth-closure gesture) compared to a meaningless one, regardless to positive or negative meaning [meaningless vs. negative *p* = 0.02; meaningless vs. positive *p* = 0.02; negative vs. positive (0.8)]. In addition, an interaction effect between factors TASK and GESTURE MOVEMENT was found [*F*_(2, 22)_ = 12.6, *p* = 0.005, $\eta_{\text{partial}}^{2}$ = 0.53, Figure 3B]. *Post-hoc* analysis revealed that participants executed the movement of lip protrusion earlier in response to the observation of a lip closure gesture, independently from its meaning and valence (mouth aperture vs. mouth closure *p* = 0.005). This difference was not significant in case of lip stretching execution (mouth aperture vs. mouth closure *p* = 0.15). All the other comparisons resulted not significant except for lip protrusion RTs measured in response to mouth aperture gestures vs. lip stretching RTs in response mouth closure gestures (*p* = 0.03).

**Figure 4:**
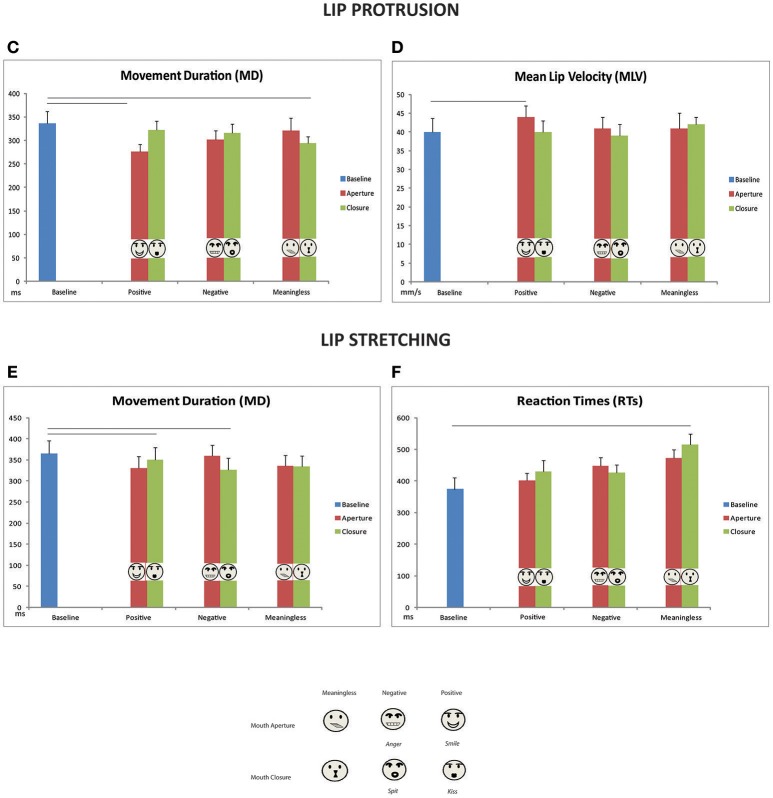
Mean values of Response Times and kinematic parameters of lip protrusion **(C,D)** and lip stretching **(E,F)** movements compared with the corresponding baseline mean value depicted at the left of each graph. Other conventions as in Figure 5.

Baseline comparisons evidenced that participants were significantly interfered when they had to execute a lip stretching movement in response to meaningless lip closure gesture [515 ms vs. 375 ms; *t*_(11)_ = 2.7, *p* = 0.04, Figure 4F].

No significant differences were found concerning lip protrusion (all contrasts resulted *p* > 0.05).

### MA

No significant effects were found for Movement amplitude.

### MD

**Figure 5:**
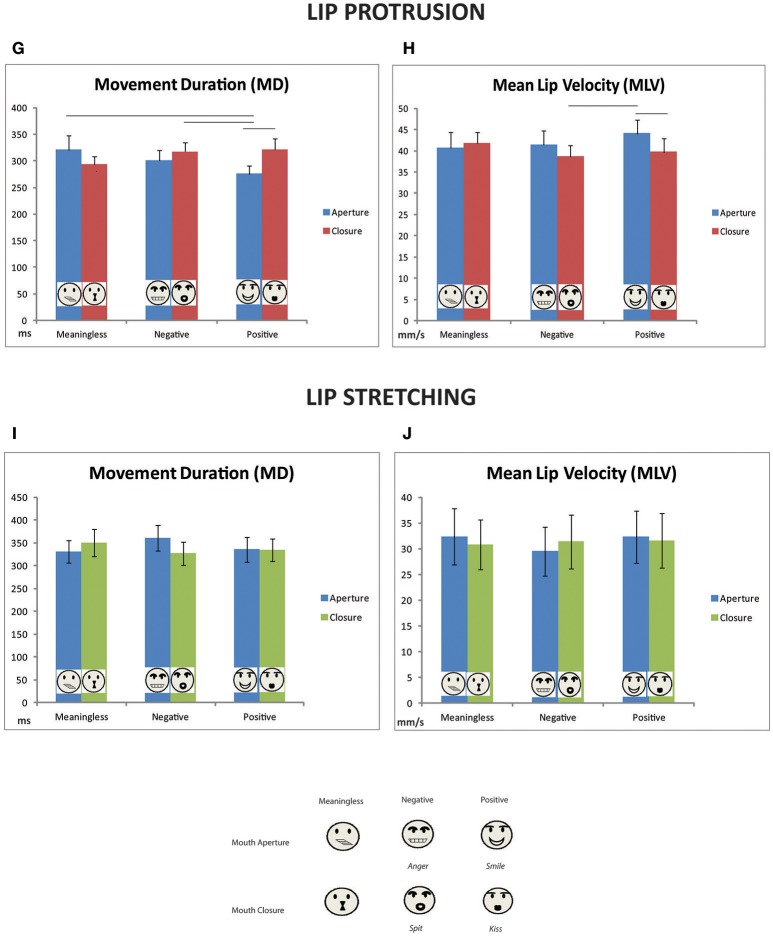
Mean values of kinematic parameters (Movement duration and Mean Lip velocity) plotted separately for lip protrusion **(G,H)** and lip stretching movements **(I,J)**. Vertical bars represent standard errors (SE). Horizontal bars represent significant difference (*p* < 0.05). The legend at the bottom depicts the corresponding stimuli for each plotted experimental condition.

Concerning MD, the ANOVA showed a significant main effect of factor TASK [*F*_(1, 11)_ = 5.2, *p* = 0.04, $\eta_{\text{partial}}^{2}$ = 0.32]: total duration of lip protrusion movement is significantly shorter compared to lip stretching. Moreover, a three-factors interaction between TASK, GESTURE MOVEMENT, and VALENCE was found [*F*_(2, 22)_ = 5.5, *p* = 0.01, $\eta_{\text{partial}}^{2}$ = 0.33, Figures 5G–I]. Concerning Lip Protrusion, *post-hoc* analysis evidenced a significant difference between mouth aperture-meaningless vs. mouth aperture-positive conditions (*p* = 0.02), between mouth-aperture/positive and mouth-closure/negative (*p* = 0.03), and between mouth-aperture/positive and mouth-closure/positive (*p* = 0.02). In sum, the lip protrusion movement had a shorter duration if executed after the observation of a positive mouth-aperture gesture (i.e., smile), compared to a mouth-closure gesture (i.e., kiss), as to a meaningless movement or a negative mouth-closure gesture (i.e., spit). No significant comparisons emerged within Lip-stretching conditions (Figures 5H–J).

Baseline comparisons confirmed that in the case of smile observation, lip protrusions were significantly facilitated in terms of shorter duration [337 vs. 276 ms; *t*_(11)_ = 3.5, *p* = 0.005, Figure 4C]. Another significant facilitation effect was found in the comparison between meaningless lip closure and baseline [337 vs. 294 ms; *t*_(11)_ = 2.7, *p* = 0.002, Figure 4D]. All the other comparisons resulted not significant.

Furthermore, in the case of mouth-closure positive or negative gesture observation (i.e., kiss and spit) lip-stretching duration was significantly shorter [baseline vs. kiss: 365 vs. 350 ms; *t*_(11)_ = 2.1, *p* = 0.05; baseline vs. spit 365 vs. 327 ms; *t*_(11)_ = 2.3, *p* = 0.03, Figure 5I].

### MLV

A significant main effect of TASK was found even for Mean lip velocity [*F*_(1, 11)_ = 5.0, *p* = 0.05, $\eta_{\text{partial}}^{2}$ = 0.31]. In addition a significant main effect of VALENCE was found [*F*_(2, 22)_ = 3.6, *p* = 0.04, $\eta_{\text{partial}}^{2}$ = 0.25]. *Post-hoc* analysis evidenced that in case of a positive gesture observation (i.e., smile or kiss) participants moved faster independently from task (i.e., lip protrusion or lip stretching). The three-factor interaction effect between TASK, GESTURE MOVEMENT, and VALENCE showed a trend to significance [*F*_(2, 22)_ = 2.9, *p* = 0.07, $\eta_{\text{partial}}^{2}$ = 0.21, Figures 5H–J]. *Post-hoc* analysis evidenced a significant difference within lip-protrusion condition between mouth-aperture/positive and mouth-closure/positive (*p* = 0.02) and between mouth-aperture/positive and mouth-closure/negative (*p* = 0.006). Confirming the results of MD, in the case of lip protrusion task participants moved faster after the observation of smile with respect to kiss and spit. No significant contrast emerged concerning lip stretching condition. Baseline comparisons evidenced that smile gestures facilitated lip protrusion movement [40 vs. 44 mm/s; *t*_(11)_ = 2.7, *p* = 0.02, Figure 4D]. All the other comparisons resulted not significant. No significant additional results were found in the comparisons with baseline for lip stretching.

## Discussion

Previous experiments have demonstrated the existence of a mechanism of *motor resonance* (Fadiga et al., 1995; Gallese, 2003; Rizzolatti et al., 2014), intended as the muscle-specific motor excitability induced by the observation of others' movements. More specifically, in the case of facial gestures this resulted in a *facial mimicry* effect, for which the muscles involved in the observed facial expressions are covertly activated (Dimberg and Thunberg, 1998; Dimberg et al., 2000; Niedenthal et al., 2001; Sato and Yoshikawa, 2007).

In a number of human and non-human primate species, the phenomenon of facial mimicry has also been utilized to explain that, in a communicative context, subjects automatically mimic emotional facial expression produced by the partner within 1,000 ms (Dimberg et al., 2000; Mancini et al., 2013a). As for example, human babies and adults show congruent facial reactions in response to dynamic facial expressions (Jones, 2009). Further, during playful episodes, both immature and adult gelada baboons have been observed to openly mimic play faces (Mancini et al., 2013b).

The results obtained on Response Times (RTs) in our experiment are therefore in line with these evidences and confirm our initial hypotheses. Indeed, participants started to move earlier when gestures were congruent (in terms of motor features) with the target movement, suggesting that a prior activation of the same motor program could facilitate the beginning of a subsequent response. This is consistent with a large amount of evidences showing how the observation of a congruent/incongruent motor sequence affects the execution of a subsequent movement (Brass et al., 2001); for an extensive review see (Heyes, 2011), modulating both perceptual and motor planning phases prior to movement execution (Deschrijver et al., 2017).

Moreover, the start of the movement was facilitated when the observed oro-facial actions conveyed an emotional valence compared to meaningless ones, confirming previous findings of a major excitability of the motor system in response to meaningful stimuli (Komeilipoor et al., 2014; De Marco et al., 2015). However, this effect was stronger for lip-protrusion compared to lip stretching movements. In this latter, faster RTs in response to congruent stimuli (mouth-aperture gestures) were found only compared to the baseline condition. A possible explanation for this result is that mouth-closing (i.e., kiss and spit) are more similar to lip-protrusion movement, whereas mouth-aperture (i.e., smile and anger) are slightly dissimilar to lip-stretching in terms of motor features (see Figure 1). The different grade of similarity between observed and executed mouth gesture in the case of lip protrusion and lip stretching conditions represents a possible limitation of this study. Indeed, motor dissimilarity could have influenced the power of facial mimicry effect in interaction with stimuli meaning: indeed, although the facilitating effect of gesture meaning was equally significant for both lip protrusion and stretching, differences concerning the specific emotion conveyed by the observed gestures (i.e., smile and anger vs. kiss and spit) may have differently affected protrusion and stretching response (see beyond).

More controversial are the results regarding the kinematic parameters (execution phase) of the movements performed by the participants. Results showed an interaction effect between valence and motor congruency associated with the temporal parameters of the lips movement (i.e., speed and duration), while no increase or reduction of the amplitude was found.

Specifically, we found an effect of *reversal of facial mimicry* mediated by the motor congruency: the movement of lip protrusion was significantly shorter after the observation of a positive mouth-aperture (i.e., smile) compared to baseline and all the other experimental conditions, except to negative and neutral mouth-closure gesture. Interestingly, this latter showed a facilitation effect that was similar in terms of movement duration to that produced by the smile observation, evidencing the interaction with valence features (see beyond). Moreover, lip protrusion in response to smile resulted significantly faster, in terms of higher mean velocity, compared to both mouth-closure positive (i.e., kiss) and mouth-closure negative gestures (i.e., spit). Although we did not find significant differences in the comparisons between conditions, partially complementary results show a similar effect of *reversal of the facial mimicry* even for the lip stretching movements. In particular, the observation of mouth closing conveying emotional expressions caused shorter lip-stretching movements compared to the baseline condition.

Thus, contrary to our expectations, kinematics results were not in line with those of Response Times: the effect of facilitation found in the phase of movement planning/preparation, and plausibly mediated by the motor congruency between the observed gesture and the pre-programmed motor task, was reversed during the execution phase. Specifically for selected meaningful gestures, kinematic parameters (speed and duration) were facilitated during the incongruent condition.

A possible explanation of this phenomenon is that, although the observation of a similar motor program could facilitate the initial activation of the lip movement, in a subsequent phase, characterized by the complete parameterization of the sequence, the two programs might compete, causing an interference and a lengthening in the actual movement execution. We suppose that even though the participants were not required to explicitly recognize the facial expression, they automatically recognize the observed stimuli, together with the emotion underlying the activation of that expression (Korb et al., 2016). It is thus possible that the sub-threshold muscles activation caused by the observation of emotional stimuli with specific mouth configurations have interacted with the pre-activation of the muscles involved in the execution of the task. This pre-activation might have facilitated the instantiation of a voluntary movement that involved similar muscles, but once the actual motor parameterization was complete, the two activations conflicted with each other (see also Boulenger et al., 2006; Dalla Volta et al., 2009). In addition, the two target movements were not conveying any emotional valence and were therefore not meaningful for communicative purposes. This lack of communicative value could free the motor commands from possible sensorimotor restrains which are typically present in more ecological conditions when two individuals freely interact.

Taking into account all these aspects, it is possible that the motor features of the observed oro-facial gestures could have interfered with task accomplishment (movement execution), slowing down similar motor programs and consequently producing a facilitation for the opposite motor patterns. Consistent with this hypothesis, we found the stronger *reversal effect of facial mimicry* for lip protrusion, whose motor characteristics are more similar to experimental stimuli (i.e., kiss and spit) compared to lip stretching, which involves a movement of elongation of the lip that is more different from the kind of mouth aperture present in smile and anger gestures (see Figure 1). Interestingly, anger produced a less strong effect of reversal and thus a minor facilitation of the lip protrusion compared to smile, probably because smile mostly modulates the bottom part of the face (i.e., zygomatic and risorium muscles), while anger is more effective in modulating the upper part (i.e., corrugator muscle, see (Rymarczyk et al., 2016).

An alternative explanation could be that, although not required, participants automatically and internally imitated oro-facial expressions that resulted similar to the instructed movement (Bisio et al., 2010; Campione and Gentilucci, 2011). This could result in an increased accuracy (and consequently, longer duration) that was expressed during movement execution, with the aim to replicate as better as possible the observed sequence. It is worth to note that greater amplitude and longer muscular activation was found recording covert facial EMG in response to the observation of congruent facial expressions (Niedenthal et al., 2001). This could be interpreted as the consequence of the tendency of the subjects to accurately reciprocate to a social signal. In support of this hypothesis, our results on RTs are in line with previous studies showing a facilitation effect in performing congruent observed movements in response to emotional facial expressions (Rauchbauer et al., 2015; Butler et al., 2016); unfortunately, no parameters related to action execution were measured in those studies. However, an increase of accuracy requirements and motor control in response to emotional cues was found in previous experiments that measures kinematic parameters of goal-directed sequences addressed to a social partner (Ferri et al., 2010; Stefani et al., 2015), and in general during motor interactions in a positive social context (Gianelli et al., 2013), for a review see (Krishnan-Barman et al., 2017).

Relevant to our results are the differential effects of the various types of emotions conveyed by the oro-facial expressions used in the experiment. First of all, the hypothesized effect of reversal of facial mimicry must be interpreted in relation to the stimuli conveying emotional valence. Indeed, the observation of mouth actions associated with emotions somehow modulates the effect of facial mimicry, and consequently, the interaction with the performed movement, while this was not observed in the case of meaningless stimuli. As consequence, the duration of lip protrusion after the observation of meaningless mouth-closure resulted comparable with the duration measured after the observation of smile. A possible reason for this is that, in case of unrecognizable or neutral stimuli, the effect of facial mimicry was weaker (Larsen et al., 2003) showing no interference with the executed lip movement.

Secondarily, faster movements in response to positive gestures confirmed that positive valence strongly modulates responses of the subjects during social interaction (Butler et al., 2016; De Stefani et al., 2016) Indeed, the shorter duration of lip-protrusion after observing a positive movement was measured in response to smile but not to anger; moreover, kiss resulted not effective as smile in speeding lip protrusion movements, evidencing a clear interaction between motor features and valence of the gesture. Instead, both negative and positive mouth-closure expressions, respectively kiss and spit, modulated the movement duration of lip stretching.

We interpreted these results in the light of the BET. Following BET (Ekman, 1992) six types of facial expressions associated with basic emotions (happiness, sadness, anger, fear, surprise, and disgust) are adaptations that have been inherited during phylogeny, and that have been selected for eliciting distinct, quick and modular behavioral effects in conspecifics. These six basic emotions share some fundamental features, among others these are: universality and modularity of their expression, distinct physiology and anatomy, quick onset and brief duration.

The perception of facial expressions associated with basic emotions (which in our studies are smile and anger) produced effect on facial muscles of the observer that were in some respect distinct from the effects produced by facial expressions associated to non-basic facial actions (e.g., spitting and kissing). Indeed, a stronger effect of positive gestures compared to negative one in modulating kinematics parameters was found for basic vs. non-basic expressions. A possibility is that, facial mimicry mechanism could be partially disambiguated between the two kinds of stimuli. Following BET, basic emotions in general caused a stronger response in conspecifics, which imply even a faster recognition of the emotional message. This mechanism could be reflected in a stronger mimicry effect for basic emotions, that in our study was reflected by empowered effects in modulating the motor response.

Another concurring factor lies in the “instrumental” features of the non-basic gestures, such as kiss and spit. This latter can be distinguished by smile and anger because they do not only convey a general emotional state, but are instrumental to the accomplishment of a specific goal toward the conspecific, who has to plan equally specific behavioral response. In other words, instrumental gesture might have a weaker effect in activating the motor systems involved in performing/perceiving the corresponding emotion, and from which the simulator implicitly infers the expresser's internal state (see Wood et al., 2016) because they focused on the communication of an action meaning rather than an emotional state.

Summing up, the present study shows how motor features and emotional valence of observed oro-facial gestures could automatically modulate voluntary motor behavior, in line with an embodied view of the cognitive processes involved in social interaction. In particular, the perception of facial cues conveying emotional meaning and the subsequent modulation of action execution are explicable through a model of cognition that takes into account aspects of the agent's body beyond the brain, and where action and (social) perception are closely interwoven. This is consistent with a huge number of data showing that action (but also speech) perception and execution share overlapping neural and bodily processes, so that simultaneous activation by observation and execution modulates behavioral performance (for reviews on this argument see (Blakemore and Frith, 2005; Galantucci et al., 2006; Caligiore and Fischer, 2013).

Moreover, these findings evidence differences in perception of basic and instrumental emotional expressions, confirming the importance of a distinction of emotional stimuli based on BET theory (Ekman, 1992), at least in an embodied cognitive framework.

Finally, this study suggest that further investigations are needed to clearly disambiguate the role of valence in relation to the kinematic features of the facial expression, eventually integrating behavioral results with measurements at electrophysiological level.

## Author contributions

AT was the main contributor for designing the experiment, but all authors participated to this processes with feedback, suggestions, and recommendations. The manuscript was mainly written by the AT and DD, with MG and PF having continuous supervisor roles. DD managed all the part of the experiment and interpretation of the results. The experiment was conducted mainly by AT, VG, and DD, while MG and PF having supervisor roles.

### Conflict of interest statement

The authors declare that the research was conducted in the absence of any commercial or financial relationships that could be construed as a potential conflict of interest.
